# Gut Microbiome-Associated Effects of Plant-Based Diets on Glucose Homeostasis, Body Composition, and Cognitive Function: A Scoping Review

**DOI:** 10.1016/j.advnut.2026.100610

**Published:** 2026-02-22

**Authors:** Colin AJ van Kalkeren, Tanja C Adam, Ellen E Blaak

**Affiliations:** 1 Department of Human Biology, Maastricht University Medical Centre+, Maastricht, The Netherlands; 2 Department of Nutrition and Movement Sciences, Maastricht University Medical Centre+, Maastricht, The Netherlands

**Keywords:** plant-based diets, vegetarian, fermentation, microbiome, SCFA, insulin resistance, obesity, neurocognition, whole grain

## Abstract

The worldwide prevalence of noncommunicable diseases, including obesity, type 2 diabetes, and neurocognitive decline, has increased rapidly over the last decades, warranting healthy and sustainable strategies to counteract these conditions. The gut microbiome is increasingly recognized as an important factor in the development of these metabolic diseases. Current nutritional guidelines advise toward more plant-based diets for health and sustainability reasons. These diets contain relatively high quantities of dietary fibers that can be fermented by the gut microbiota in the colon. This produces short-chain fatty acids (SCFA), recognized for their beneficial effects on insulin resistance, inflammation, and satiety. In the absence of fermentable fibers, however, colonic proteolytic fermentation increases, producing metabolites that are potentially harmful to host health. Moreover, plant-based proteins are less digestible compared with animal-based proteins, potentially increasing proteolytic fermentation. However, the extent to which plant-based diets affect the microbiome, and thereby host health, is still unknown. We, therefore, conducted a scoping review investigating the effects of plant-based dietary interventions on the microbiome in relation to the effects on body composition, glucose metabolism, and neurocognition. Overall, (healthy) plant-based diets, either as a whole dietary pattern, whole-grain consumption, or plant-protein-rich products, can contribute to lower body (fat) mass, fasting glucose, improve insulin resistance and episodic memory, and reduce psychological distress and anxiety. Notably, no adverse effects of plant-based diets were reported in any of the studies. The studies that reported alterations to microbial composition after plant-based interventions generally show increased microbial diversity and abundance of SCFA-producing species such as *Prevotella*, *Lachnospiraceae,* and *Bifidobacteria.* However, measures on microbial functionality, based on microbial metabolites such as SCFA and branched-chain fatty acids, were often lacking or remained mostly unaffected. Furthermore, research on the long-term and individual effects of both healthy and unhealthy plant-based diets on host health and the microbiome is required.

**Statement of significance**

This review is the first to assess the effects of plant-based diets on body composition, glucose homeostasis, and neurocognition, in light of changes within the gut microbiome, showing convincing benefits for both metabolism and the microbiome.

## Introduction

The global rise in overweight, obesity, and related chronic noncommunicable diseases, such as type 2 diabetes mellitus (T2D), cardiovascular disease (CVD), and mental health problems, is posing a growing burden on individuals, healthcare systems, and economies worldwide. Apart from many lifestyle, environmental, and genetic factors, an imbalance in microbial communities in the human gut has been implicated in the etiology of these chronic disorders [1]. During the last decades, the impact of the human diet on host health and metabolism, as well as on microbiome composition and functionality, has become more recognized [[1], [2], [3], [4]]. Dietary quality, in the context of different food sources and dietary patterns, has emerged as a key modulator of metabolism and health outcomes [[5], [6], [7], [8], [9], [10], [11], [12]]. Plant-based dietary patterns, for example, have been associated with a reduced risk of developing CVD, T2D, and certain types of cancer, as well as improved mental well-being, while also contributing to more sustainable food growing and consumption [[13], [14], [15], [16], [17]]. As a result, dietary guidelines advocate a shift from an animal-based to a more plant-based diet [7,8,[10], [11], [12]]. However, even though these diets have been recognized for their metabolic benefits for host health, it is unclear how these diets mechanistically affect host health, and to what extent modulation of the composition and functionality of the gut microbiome plays a tangible role herein.

Plant-based diets generally consist of a variety of fruits, vegetables, legumes, and nuts, and contain many vitamins, antioxidants, unsaturated fatty acids, indigestible proteins, and dietary fibers [14,18]. Moreover, they ordinarily lack an abundance of simple carbohydrates and energy-dense substances. However, there are many plant-based dietary regimens to be distinguished. Vegan diets exclude all animal-derived products; vegetarian diets generally exclude meat but may include dairy (lacto-vegetarianism), eggs (ovo-vegetarianism), both dairy and eggs (lacto-ovo–vegetarianism), and potentially fish (pesco-lacto-ovo–vegetarianism). Furthermore, diets can be classified based on their geographic origin, such as the Mediterranean diet (MD) or Atlantic diet (AD) [14]. The MD is characterized by high amounts of plant-derived foods (fruit, vegetables, legumes, PUFA-rich vegetable oils, and minimally refined cereals), moderate dairy consumption, and low red meat intake [14]. The AD is similar to the MD, but generally contains more fish and red meat [19]. The diversity in definitions of these plant-based diets means there is also a large variety in expected metabolic effects, with some diets being more healthy and others considered rather unhealthy plant-based diets. Plant-based diets generally contain higher amounts of dietary fibers, plant-based proteins, and phytochemicals such as tannins, phenols, indoles, and flavonoids, recognized for their important role in improving or maintaining a healthy metabolic status. However, there are plant-based dietary regimens that are considered unhealthy, containing refined grains, sugary beverages, and sweets [7,14,[20], [21], [22]].

A recent review investigated the differences between animal- and plant-based diets in terms of energy and macronutrient intake and found that, in general, plant-based diets, compared with omnivorous diets, led to lower or similar energy, protein, saturated fat, and MUFA intake, whereas PUFA intake was higher [14]. A higher proportion of people consuming plant-based diets, however, ingested amounts of protein that were well below their acceptable range (<0.6 g/kg/d or <10–20 E%/d), contributing to the concerns of sufficient macronutrient intake when consuming plant-based diets [14]. Besides, both the digestibility and quality of plant-derived proteins, compared with animal-based proteins, are lower, potentially leading to (essential) amino acid deficiencies and loss of muscle mass, especially in sarcopenia-prone individuals [23,24]. Although plant-proteins may aid gastrointestinal bulk formation and satiety, they may also induce proteolytic fermentation, producing rather detrimental substrates as will be discussed below [[25], [26], [27]]. Additionally, more unhealthy plant-based diets have been associated with increased morbidity and mortality, compared with healthy plant-based alternatives, warranting a deeper understanding of what healthy plant-based diets and their metabolic effects entail [21,22].

We recently proposed that the balance of colonic fermentation of dietary fibers (saccharolytic fermentation) and proteins (proteolytic fermentation) is an important determinant of cardiometabolic health, with a switch toward saccharolytic fermentation providing favorable health effects [26,28,29]. Saccharolytic fermentation yields beneficial substrates, such as short-chain fatty acids (SCFA). More specifically, studies have shown that distal, and not proximal, colonic SCFA availability could induce systemic benefits [30,31]. However, the effects of plant-protein-rich diets on the human gut microbiome, as well as the microbiome-associated effects of these diets on glucose homeostasis, body weight control, and neurocognitive function, are not yet thoroughly understood. Although increasing the intake of these plant-based foods poses a beneficial effect on human health and the environment, concerns have been raised about the impact of diets high in (plant-derived) proteins on colonic mucosa and microbial composition, due to increased proteolytic fermentation [32], but also for their lower protein quality, among others. This review therefore aims to assess the interaction of the human gut microbiome with plant-based diets and the effects on host health, focusing on glucose metabolism, body composition, and neurocognitive function. Thereby, we aim to contribute to the understanding of the interaction between the microbiome and plant-based diets and their role in the development of obesity, insulin resistance, and neurocognitive dysfunction, posing new insights on more preventive and curative strategies for these increasingly prevalent disorders, while incorporating the potential downsides of (unhealthy) plant-based diets.

### The gut microbiome and plant-based diets

The gut microbiome, being highly adaptable and affected by many factors, including dietary habits, is a major mediator in many metabolic processes, including the fermentation of dietary substrates [1,33,34]. Because the human gut microbiome consists of trillions of different bacteria, viruses, and yeasts, a detailed overview of the microbiome in relation to health and disease is beyond the scope of this review. We will, however, introduce some of the commonly studied and affected microbes related to plant-based dietary interventions.

Although the microbiome is unique per individual, microbial composition and functionality may differ between healthy and metabolically compromised individuals. A differential microbial composition may potentially explain (non)response to dietary interventions [[35], [36], [37]]. Alpha and beta diversity are found to differ between people with a healthy weight and individuals with obesity [[38], [39], [40], [41]]. Alpha diversity, which can be increased through physical activity and dietary fiber intake, has been negatively associated with weight gain throughout life [[37], [38], [39], [40], [41], [42]]. Similarly, the Firmicutes/Bacteroidetes ratio, although a heavily debated marker with no evidence on the causality for diabetes or obesity, has been negatively associated with metabolic status [[35], [36], [37]].

Few studies have assessed the effects of plant-based diets on the microbiome. A recent review aimed to summarize the changes in microbial composition after plant-based dietary regimens, showing distinct microbial profiles between vegetarian and omnivorous diets [43]. Vegan/vegetarian diets are associated with a higher abundance of *Clostridia*, *Bacteroidia*, *Lanchnospiraceae*, *Ruminococcaceae*, *Bacteroides*, *Roseburia*, *Akkermansia*, *Faecalibacterium prausnitzii*, and *Bacteroides fragilis* [43,44]. Besides, abundance of *Desulfovibrionaceae*, *Faecalibacterium*, *Parabacteroides*, and *Eubacterium rectale* was lower [43,44]. Additionally, plant-based dietary habits have been positively associated with *Prevotella* and *Bacteroides* abundances, and with reduced relative *Firmicutes* abundance [[44], [45], [46], [47]]. Similarly, the MD has been associated with increased *Prevotella* and *Bifidobacterium* abundance, an increased *Prevotella/Bacteroides* ratio, as well as decreased Firmicutes/Bacteroidetes ratios [44,[48], [49], [50]]. These MD-related microbial species may be driven by the high plant-based products within these diets, although fish sources and omga-3 (n–3) fatty acids have been linked to increased *Bifidobacterium*, *Akkermansia*, *Lachnospiraceae*, *Roseburia*, and *Prevotella* abundance as well in both humans and rodents [[51], [52], [53]]. In line with the MD, higher AD adherence has also been linked to increased *Bifidobacterium* abundance, which in turn increased with lower BMI in healthy adults [54]. The above primarily depicts beneficial results related to rather healthy plant-based diets. However, unhealthy plant-based diets have been linked to lower alpha diversity and lower abundance of bacterial species associated with metabolic benefits, such as *Faecalibacterium prausnitzii* [55].

Microbial functionality, besides microbial composition, plays an important role in metabolic health as well. Saccharolytic fermentation, for example, the fermentation of complex carbohydrates such as dietary fibers and resistant starches, produces SCFA once these dietary substrates enter the colon. Dietary fibers are nondigestible, plant-derived polysaccharides found in vegetables, fruits, and legumes [18,44]. They can be classified as soluble and insoluble, which determine the extent, rate, and location of microbial fermentation and the induction of microbial and metabolic alterations [56]. Soluble fibers are generally fermented quite rapidly [57,58], whereas insoluble fibers support gastrointestinal transit and contribute to a smooth luminal transport of chyme [59,60]. Both types have been found to alter microbial composition in vitro [[61], [62], [63]] and in vivo [64,65]. Both fibers can reduce the relative abundance of Firmicutes and increase Bacteroidetes abundance [[66], [67], [68]], while increasing SCFA production [[66], [67], [68], [69]]. These SCFA, rapidly produced in the proximal colon and primarily consisting of acetate, propionate, and butyrate, have been recognized for their benefits to host health [34,70]. They affect a broad range of metabolic processes, improve colonocyte functioning and gut barrier integrity [26,29], affect the production of satiety hormones (peptide YY) and glucagon-like peptide 1 (GLP-1) [26,29], and contribute to alleviation of multiorgan insulin resistance, enhanced energy expenditure, increased pancreatic β-cell function, and improved lipid oxidative capacity, while reducing systemic inflammation [26]. Besides, they may affect food-reward-related brain activity and contribute to learning and cognitive function [71]. Examples of SCFA-producers that are more commonly found in healthy, compared with metabolically compromised individuals, are bacteria belonging to the *Bifidobacteria*, *Prevotella, Roseburia*, and *Ruminococca* genera [34,70], which may be upregulated after fiber consumption [56].

Although whole food, plant-based products, such as whole-grain bread and pasta, vegetables, fruit, and legumes, are rich in dietary fibers, the presence of indigestible plant-proteins may contribute to increased proteolytic fermentation. This generally occurs in the absence of fermentable carbohydrates and primarily in the transverse and distal colon, producing substrates such as branched-chain fatty acids (BCFA), ammonia, indoles, p-cresol, methane, and hydrogen sulfate. Even though indoles have also been linked to supporting intestinal barrier function and antioxidative effects [72], many of these substances are believed to have a detrimental effect on the human body [26,[73], [74], [75]]. They are found to increase low-grade inflammation and reduce insulin sensitivity, intestinal barrier function, and lipid buffering and lipid oxidative capacity, and have been associated with colorectal cancer [26,[73], [74], [75]]. Evidence on the microbiome-altering effects of plant-proteins remains limited and inconclusive [76,77]. Although animal-protein intake has been associated with increased beta diversity after an animal-based diet in 1 study, both alpha and beta diversity were not affected by plant-protein consumption within a vegetarian diet [78]. Similarly, several other studies were unable to show changes in microbial composition after plant-based protein consumption, whereas animal-based proteins significantly changed microbial abundances [76,[79], [80], [81]]. Besides, animal-based proteins were positively associated with *Bacteroides*, which are partly recognized for their BCFA-production, *Alistipes,* and *Bilophila* abundances, and negatively associated with *Roseburia* and *Eubacterium rectale* abundances, although these effects were primarily observed after isolate supplements and not within whole-diet approaches [34,76,[82], [83], [84], [85]]. Aside from affecting microbial composition itself, protein intake also affects luminal and circulating compounds derived from microbial metabolism, such as trimethylamine (TMA) [86,87]. TMA is a precursor of trimethylamine-N-oxide (TMAO), which is generally derived from animal-based proteins [86,87], and has been linked to CVD. Although TMAO can be formed from plant-proteins as well, TMA oxidation in the context of a plant-based diet is significantly lower compared with carnivorous diets [86,88].

Besides complex carbohydrates and proteins, dietary fats and phytochemicals should not be overlooked in the context of microbiome-altering effects, although their effects are less studied and remain inconclusive. Many studies investigating the fat-induced effects on the microbiome primarily include omnivorous diets or animal-based interventions [76,[89], [90], [91], [92], [93], [94], [95]], preventing direct extrapolation to the effects of plant-based diets, as plant-based fats are generally less saturated and frequently rich in MUFAs and PUFAs [92]. A high-fat omnivorous diet has been associated with decreased *Faecalibacterium* and increased *Alistipes* and *Bacteroides* abundance in healthy adults [76,95], whereas a low-fat omnivorous diet in individuals with overweight increased *Bifidobacterium* abundance [90]. Saturated fats have been associated with increased abundances of *Faecalibacterium prausnitzii* and *Proteobacteria*, whereas MUFA-rich may increase butyrate-producing bacteria [76,90,91]. PUFAs have been linked to reduced Firmicutes/Bacteroidetes ratios and increased *Bacteroides*/*Prevotella* ratios, as well as increased *Coprococcus*, *Lachnospiraceae*, *Bifidobacterium*, and *Bacteroides spp* abundance [76,89,93,94,96].

Phytochemicals, plant-derived substrates recognized for their metabolic benefits, include polyphenols. These polyphenols, consisting of lignans, flavonoids, and tannins derived from fruits and seeds, among others, are linked to many positive health effects. These effects include protection against systemic inflammation, improving insulin resistance, and lowering blood pressure [86,88,[97], [98], [99], [100]], and protection against diseases such as CVD, T2D, and different types of cancer [[101], [102], [103], [104]]. Many of these effects are related to the interaction between the phytochemicals and the microbiome, potentially through microbial conversion into biologically active compounds [44,101,[104], [105], [106], [107]]. Besides, they have been found to affect microbial composition, bacterial growth, and cross-feeding, phytochemicals to increased abundance of *Lactobacillus, Bifidobacterium, Prevotella, Blautia coccoides-Eubacterium rectale, Bacteroides,* and *Proteobacteria* [108,109].

Overall, the microbiome can be altered by plant-based dietary regimens, with dietary fibers found to be the most potent mediators, although this heavily depends on fiber type and dose, as well as the differences in microbial composition and fiber-degrading capacities between metabolic phenotypes [56,[110], [111], [112], [113], [114]]. Even though there are great variations in responses to plant-based regimens, they generally seem to lead to increased abundances of microbes that are associated with beneficial traits [1,13,14,56,115,116]. However, although nutritional interventions can likely benefit metabolic health as well as alter microbial communities, whether these metabolic improvements through plant-based diets can be attributed to changes in microbial profiles will be explored.

## Methods

### Literature search and data extraction

This review was conducted using an online systematic search for each of the 3 subdomains using PubMed, of which all search strings can be found in the supplementary data. Results up to 31 December, 2025, are included in this review. Only original papers on (randomized) intervention, cross-sectional, observational, or cohort studies related to plant-based diets or individual components that are part of a plant-based diet were included, such as whole-grain products. Filters were used to select papers only with full text available in either English or Dutch, conducted in human adults. Reviews, meta-analyses, conference publications, chapters, case reports, letters, commentaries, and other nonoriginal studies were omitted from this review. All titles and abstracts were screened for eligibility by 2 researchers (CAJvK and TA) in an independent manner. Eligibility was defined as any title or abstract referring to plant-based diets or products that investigated one of the subdomains and contained information on microbial composition or functionality. Studies focusing on specific diseases or only specific dietary supplements/prebiotics/probiotics that are added to a regular diet, investigating medicinal treatments, study protocols, studies in children or rodents, in vitro studies, and papers without data on the microbiome or 1 of the investigated health domains were omitted from the reviewing process. No conflicts in paper selection occurred between the researchers. After selection of the papers that met the eligibility criteria, CAJvK reviewed the full texts and extracted data related to the topic of this review, including measures of body composition (including body weight, body fat distribution [waist circumference (WC), hip circumference (HC), waist-hip-ratio (WHR), fat mass, fat-free mass, visceral fat mass (VAT)], markers of glucose homeostasis [fasting or postprandial plasma glucose or insulin concentrations, hemoglobin A_1c_ (HbA_1c_), the HOMA-IR, and/or the Matsuda Index], data related to neurocognitive performance or diseases (including executive functioning, attention, psychomotor skills, memory, depression, dementia), and data on microbial composition and functionality.

In general, all strings contained Medical Subject Headings terms and synonyms, and open terms, including but not limited to “Plant-based diets,” “Plant-based,” “Plant diets” AND “Microbiome,” “Microbiota,” and “Microbial composition” AND either (“Insulin resistance,” “Glucose homeostasis,” “HOMA-IR,” “Diabetes mellitus” etc.), (“Body composition,” “Body weight,” “BMI,” “WHR,” “Waist circumference,” and “fat mass,”) or (“Stress,” “neurocognitive functioning,” “neurocognition,” “brain,” and “cognition.”) The complete search strings can be found in the supplementary material.

### Search results

All 3 searches provided sufficient amounts of studies to be incorporated into this review. The “body composition” search resulted in 82 papers, of which 59 were excluded (49 based on title and abstract, and an additional 10 after full-text appraisal), leaving 23 papers to be included (Table 1 and Supplementary Figure 1). The “glucose homeostasis” search yielded 63 results, of which 38 were excluded based on title and abstract. After full-text assessment, an additional 9 papers were excluded, resulting in 16 studies being included in this review (Table 2 and Supplementary Figure 2). The “Neurocognition” search string provided 48 papers, of which 35 were excluded based on title and abstract, and another 8 papers were excluded after full-text revision (Table 3 and Supplementary Figure 3).

**TABLE 1:** An overview of the included studies with a summary of the plant-based dietary effects on body composition and the gut microbiome

Author, y	Type of study	Study population	Study location	*n*	Intervention	Control	Body composition outcomes	Microbiome outcomes
Ahrens et al., 2021 [117]	Open-label single-group intervention	M/F, 18–99 y	United States	73	In-house nutritional education program; 100% plant-based whole foods.	—	BW/BMI ↑. WC/HC ↓.	Alpha diversity ↑. *Lachnospiraceae/Ruminococcaceae* ↑. *Bacteroides* ↓.
Ampatzoglou et al., 2015 [118]	RCT, crossover	Healthy M/F, 40–65 y, BMI 20–35	United Kingdom	33	>80 g/d whole grain.	<16 g/d whole grain.	=	=
Barkhidarian et al., 2025 [119]	Cross-sectional	Healthy women in luteal phase, 18–50 y, BMI 20–35	Iran	91	—	—	Unhealthy PD adherence (-) FFM.	↑ (healthy) PD adherence: (+) *Prevotella/A. Muciniphila*, (-) Firmicutes.
Basciani et al., 2020 [120]	RCT, open-label	Healthy M/F, 50–70 y, BMI 30–40, HOMA-IR ≥2.5	Italy	48	Whey compared with animal compared with vegetable protein; all within VLCKD.	—	Whey/vegetable protein: BW ↓. All: WC ↓. Vegetable/animal protein: HC ↓.	All proteins: Firmicutes ↓ (animal < vegetable and whey). Whey/animal protein: Bacteroidetes ↑.
Bowen et al., 2019 [121]/Choo et al., 2019 [122]	RCT	M/F, 20–70 y, BMI >26.9, elevated WC/FBG, no pharmacologically-treated T2D	Australia	76	56 g/d almond	Biscuit (isocaloric).	=	Almond vs. CON: - Richness, evenness, diversity ↑ - *Ruminococcaceae* ↑ - fSCFA =
Burns et al., 2025 [123]	RCT	Healthy M/F, 18–65 y, BMI <25	United Kingdom	46	Plant-based FMD-LP; plant-based FMD-HP.	Isoenergetic, habitual diet.	FMD: BM, FFM, FM ↓; CON =. FMD-HP: VAT ↓; FMD-LP/CON =	Alpha diversity: FMD-HP ↑; FMD-LP/CON =. Firmicutes abundance: FMD-LP ↓; FMD-HP ↑; CON =
Chang et al., 2025 [124]	RCT, crossover	M/F, 20–64 y, BMI 27–35/elevated WC/FBG 5.5–6.9 mmol/l	Taiwan	16	50 g/d protein-rich foods, 30.4 g/d black soymilk, 95 g/d cooked low-fat pork (21 g protein).	73.5 g low-fat ground pork (15 g protein), 166 g/d pork tenderloin (34 g protein).	=	Alpha/beta diversity: =. *Butyricicoccus*/*Rothia*: ↑ in intervention group.
Christensen et al., 2019 [125]	RCT	Healthy M/F, BMI >25	Denmark	70	Whole-grain wheat, whole-grain rye.	Refined wheat.	—	Low P/B (+) low BMI. Prevotella (-) Δbody weight in whole-grain group. Baseline *Prevotella* (+) weight loss.
Cooper et al., 2017 [126]	RCT	Healthy M/F, 19–46 y, BMI 20–28, low grain consumers	United States	46	Whole grain.	Refined grain.	=	*Akkermansia*/*Lactobacillus* abundance: whole grain ↑, refined grain ↓.
Eid et al., 2015 [127]	RCT, crossover	Healthy M/F, 18–55 y, BMI 20–25	United Kingdom	22	50 g/d Ajwa dates	Maltodextrose, sugar-content matched.	=	=
Foerster et al., 2014 [128]	RCT, crossover	Healthy M/F, 20–60 y	Germany	20	Whole grain (40 g fiber/d), <30 g/d red meat.	200 g/d red meat, minimal fiber.	Whole-grain: BMI/FM/BW ↓. CON: =	Whole grain: diversity ↑. SCFA: =
van Kalkeren et al., 2025 [129]	RCT	M/F 30–75 y, BMI 28–40, prediabetes	Netherlands	40	Fiber mixture + plant-protein-rich diet.	Maltodextrin + plant-protein-rich diet.	=	Microbial composition: =. Plasma BCFA: Fiber =/↓; CON ↑ (IsoVA). Fecal/plasma SCFA, fecal BCFA: =
Karl et al., 2017 [130]	RCT	Healthy M/F (menopausal), 40–65 y, BMI 25–35	United States	81	Whole grain.	Refined grain.	=	Lower alpha diversity at baseline in whole-grain group, but not postintervention. Beta diversity was different preintervention, but not postintervention. SCFA: whole grain =; refined grain ↓.
Ribeiro et al., 2024 [131]	RCT	Healthy M/F, 65–75 y	Australia	113	Ad libitum diets: OH-fat, OH high-CHO (OHC), semi-VH-fat, semi-VH-CHO.	—	BW ↓ in all groups, but primarily after semivegetarian diets.	OHC: diversity/Proteobacteria ↑. Microbial diversity (-) weight loss. Plasma acetate ↑ in all groups, but primarily in VHC-group.
Roager et al. 2019 [132]	RCT	M/F, 20–65 y, BMI 25–35, elevated WC or SBP/FBG 6.1–6.9/low HDL	Denmark	50	75 g/d whole grain	<10 g/d whole grain.	Whole grain: BW, SAD ↓.	Whole grain: *F. prausnitzii/P. copri* ↑, *B. thetaiotaomicron* ↓. CON: *F. prausnitzii/P. copri* ↓, *B. thetaiotaomicron* ↑. Whole grain/fiber intake: (+) *Clostridiales/F. prausnitzii*, (-) *B. thetaiotaomicron.*
Rodríguez-Lara et al., 2022 [133]	Cross-sectional	M/F, 18–25 y	Mexico	50	—	—	—	*C. coccoides-E. rectale* ↓ in individuals with overweight. Fiber/energy/protein/CHO intake (-) Bacteroidetes in individuals with normal weight.
Rosas et al., 2023 [134]	RCT	Healthy M/F, 20–35 y	United States	20	42 g/d nut mixture	Potato chips (isocaloric, 46 g/d).	=	Alpha/beta diversity: = Microbial composition: =
Schutte et al., 2018 [135]	RCT	M/F (postmenopausal), 45–70 y, BMI 25–35, cholesterol >5.0 mmol/l	Netherlands	50	98 g/d whole-grain wheat	98/d refined wheat.	=	Alpha diversity: whole grain =; CON ↓.
Shi et al., 2024 [136]	Cross-sectional	M/F, 18–80 y	China	2998	—	—	Nut consumption (-) WC/WHR/android FM.	Alpha diversity: (+) nut consumption/GF%; (-) AF%/AGR/WC/WHR. Beta diversity differed between high and low-nut consumers. Anaerobutyricum: ↑ in high nut consumers, (-) WHR/AF%/AGR.Anaerotaenia: (-) AF%/AGR/WC/WHR, (+) GF%. Fusobacterium: ↑ in low-nut consumers, (+) WC/WHR/AF%/AGR, (-) GF%.
Vitaglione et al., 2015 [137]	RCT	Healthy M/F, >18 y, BMI 25–35, no habitual whole-grain cereal consumption	Italy	68	70 g/d whole-grain biscuits	33 g/d refined-grain crackers and 27 g/d toasted bread.	=	Whole-grain: *Prevotella* ↑, *Dialister/Bifidobacterium/Blautia/Collinsella* ↓.
Wang et al., 2021 [138]	RCT	M/F, 20–65 y, elevated WC + one risk factor for MetS	China	209	56 g/d peanuts	Rice bars (isocaloric, 82 g/d).	Peanut: WC ↓.	=
Zhao et al., 2024 [139]	RCT	M/F, 18–65 y, T2D and elevated cholesterol	China	68	gBrR, gBlR.	White rice.	GBR: Fat% ↓ compared with white rice.	gBlR and CON: *Desulfobacterota* ↓. gBlR compared with gBrR: *Fusobacteria* ↑. CON compared with GBrR: *Clostridium_sensu_stricto_1* and *Veillonella* ↑. gBlR and gBrR compared with CON: *Alloprevotella* ↑.

Positive associations indicated by (+), negative associations by (-), upregulation/increase by ↑, downregulation/decrease by ↓, no changes by =. When there are differential effects between groups, group names are included in the results.

Abbreviations: AF, android fat; AGR, Android-gynoid-ratio; BW, body weight; CHO, carbohydrate; CON, control; FBG, fasting blood glucose; FFM, fat-free mass; FM, fat mass; FMD, fasting-mimicking diet; fSCFA, fecal SCFA; gBlR, germinated black rice; gBrR, germinated brown rice; GF, gynoid fat; HC, hip circumference; HP, high-protein; IsoVA, isovaleric acid; LP, low-protein; MetS, metabolic syndrome; OH, omnivorous high; P/B, Prevotella/Bacteroides ratio; PD, plant-based diet; RCT, randomized controlled trial; SAD, sagittal abdominal diameter; SBP, systolic blood pressure; SCFA, short-chain fatty acids; T2D, type 2 diabetes; VAT, visceral adipose tissue; VH, vegetarian high; VLCKD, very low-caloric ketogenic diet; WC, waist circumference; WHR, waist-hip-ratio.

**TABLE 2:** An overview of the included studies with a summary of the plant-based dietary effects on glucose homeostasis and the gut microbiome

Author, y	Type of study	Study population	Study location	*n*	Intervention	Control	Glucose homeostasis outcomes	Microbiome outcomes
Basciani et al., 2020 [120]	RCT, open-label	Healthy M/F, 50–70 y, BMI 30–40, HOMA-IR ≥2.5	Italy	48	Whey vs. animal vs. vegetable protein; all within VLCKD.	—	Vegetable protein: insulin/HOMA ↓ Whey/animal protein: FBG/insulin/HOMA ↓	All proteins: Firmicutes ↓ (animal < vegetable and whey). Whey/animal protein: Bacteroidetes ↑.
Bel Lassen et al., 2021 [140]	Observational	M/F Cohort 1: Surinamese, Ghanaian, Turkish, Moroccan, and Dutch adults Cohort 2: 8 groups of different metabolic phenotypes, some with cardiac comorbidities	Netherlands, France, Germany, Denmark	1759/1549	—	—	Protein intake: (+) pre-T2D and T2D, only explained by animal protein and not plant-protein consumption.	Alpha diversity: (+) fat intake, (-) CHO intake, (=) protein intake. Beta diversity: (+) fiber and plant-protein intake.
Chang et al., 2025 [124]	RCT, crossover	M/F, 20–64 y, BMI 27–35/elevated WC/FBG 5.5–6.9 mmol/L	China	16	50 g/d protein-rich foods, 30.4 g/d black soymilk, 95 g/d cooked low-fat pork (21 g protein).	73.5 g low-fat ground pork (15 g protein), 166 g/d pork tenderlon (34 g protein).	=	Alpha/beta diversity: = *Butyricicoccus/Rothia*: ↑.
Ding et al., 2022 [141]	RCT	M/F, 18–70 y, T2D	China	112	Germinated brown rice (gBrR)	White rice	gBrR: FBG/HbA_1c_ ↓.	GBrR: fSCFA ↑, *Bifidobacterium/Actinomycetes/Pasteurellales* ↑. CON: *Bifidobacterium* ↓.
Eriksen et al., 2020 [142]	RCT	Men, 49–74 y, MetS	Sweden	49	Whole-grain rye	Whole-grain wheat	=	Rye vs. wheat: *Bifidobacterium/Lachnospira/Butyricoccus* ↓, fecal butyrate ↑.
Galié et al., 2021 [143, 144]	RCT	M/F, 25–60 y, BMI 25–35, MetS and non-MedDiet.	Spain	44	Mixed nuts + nonMedDiet	MedDiet	MedDiet: FBG/insulin/HOMA ↓.	MedDiet: (+) *Lachnospiraceae*.
van Kalkeren et al., 2025 [129]	RCT	M/F 30–75 y, BMI 28–40, prediabetes	Netherlands	40	Fiber mixture + plant-protein-rich diet	Maltodextrin + plant-protein -rich diet	IS: fiber ↓; placebo ↑. Glucose, insulin, HbA1c: =	Microbial composition: =. Plasma BCFA: fiber =/↓; placebo: IsoVA ↑. Fecal/plasma SCFA, fBCFA: =
Medina-Vera et al., 2019 [145]	RCT	*T2D*: M/F, 30–60 y, BMI 25–40, pharmacologically-treated T2D.	Mexico	0	Mix of plant-protein sources (200 kcal).	Caseinate+maltodextrin (isocaloric).	Plant-proteins: glucose/HbA_1c_ ↓.	Plant-protein consumption: *F. Prausnitzii/A.Muciniphila/B. longum/B. fragilis*/alpha diversity ↑, *P. Copri* ↓.
Neascu et al., 2025 [146]	RCT, crossover	M/F 18–65 y, healthy, BMI 18–35.	United Kingdom	20	80 g/d buckwheat	80 g/d fava bean	Fava: FBG/insulin ↓.	Buckwheat: *Roseburia/Anaerostipes/Bifidobacteria* spp ↓. Fava: *Lactobacillus ↓, Coprococcus/Bifidobacterium* ↑. fSCFA: =.
Roager et al., 2019 [132]	RCT	M/F, 20–65 y, BMI 25–35, elevated WC or SBP/FBG 6.1–6.9/low HDL	Denmark	50	75 g/d whole grain	<10 g/d whole grain	=	Whole grain: *F. prausnitzii/P. copri* ↑, *B. thetaiotaomicron* ↓. CON: *F. prausnitzii/P. copri* ↓, *B. thetaiotaomicron* ↑. Whole-grain/fiber intake: (+) *Clostridiales/F. prausnitzii*, (-) *B. thetaiotaomicron.*
Stefani et al., 2018 [147]	Cross-sectional	Healthy women 19–50y	Indonesia	240	—	—	Healthy eating index: (+) FBG/HbA_1c._	=
Su et al., 2022 [148]	RCT	M/F, >18 y, T2D	China	16	Diet (whole grains, probiotics, prebiotics)	Diet+fecal transplant^1^	Diet: HbA_1c_/FBG ↓, glycemic control ↑.	Diet: *Prevotella* became most abundant (replacing *Bacteroides*), *Bifidobacterium*/*Blautia* ↑, *Roseburia/Ruminococcus* ↓. *Bifidobacterium*: (+) glycemic control. *Lactobacillus*/*Collinsella*/*Neisseria:* (-) FBG. *Desulfovibrio*: (+) FBG.
Tuccinardi et al., 2019 [149]	RCT, crossover	M/F, BMI>30	Israel	10	Walnuts (48 g/d)	Isocaloric safflower smoothie.	Walnut: glucose/insulin responses ↓.	Walnuts: fBCFA ↓.
Wu et al., 2025 [150]	RCT	M/F 45–75 y, BMI 19.5–32, prediabetes, Chinese	Singapore	127	Plant-based-rich diet, including whole grains	Meat, high-glycemic index, rice/noodles, refined grains	Plant-based diet: temporal HbA_1c_ ↓.	Plant-based diet: *Clostridia*/*Bifidobacteria* ↑, *Ruminococcus*/*Bacteroides*/*Bilophila* spp ↓. Fiber intake: (+) *E. rectale/Bifidobacterium*, (-) *Ruminococcus* spp.
Zhao et al., 2024 [139]	RCT	M/F, 18–65 y, T2D + elevated cholesterol	China	68	gBrR, gBlR	White rice	gBrR/gBlR: FBG ↓.	gBlR and CON: *Desulfobacterota* ↓. gBlR vs. gBrR: *Fusobacteria* ↑. CON vs. GBrR: *Clostridium_sensu_stricto_1* and *Veillonella* ↑. gBlR and gBrR vs. CON: *Alloprevotella* ↑.

Positive associations indicated by (+), negative associations by (-), upregulation/increase by ↑, downregulation/decrease by ↓, no changes by =. When there are differential effects between groups, group names are included in the results.

Abbreviations: CHO, carbohydrate; CON, control; fBCFA, fecal branched-chain fatty acids; FBG, fasting blood glucose; fSCFA, fecal short-chain fatty acids; gBlR, germinated black rice; gBrR, germinated brown rice; HbA1c, glycated hemoglobin A1; IS, insulin resistance; MedDiet, Mediterranean diet; MetS, metabolic syndrome; RCT, randomized controlled trial; SBP, systolic blood pressure; T2D, type 2 diabetes; VLCKD, very low-caloric ketogenic diet; WC, waist circumference.

1 Not included in further analysis due to discordance with review methods.

**TABLE 3:** An overview of the included studies with a summary of the plant-based dietary effects on neurocognition and the gut microbiome

Author (y)	Type of study	Study population	Study location	*n*	Intervention	Control	Neurocognitive outcomes	Microbiome outcomes
Haskell-Ramsay et al., 2023 [151]	RCT, crossover	Healthy M/F, 18–49 y, BMI 18.5–30	United Kingdom	79	30 g/d nut mixture + 1 cellulose capsule	2 cellulose capsules	Nuts: PR and RVIP accuracy/PR RT ↑, peg and ball errors/numeric working memory RT ↓.	Nut: *Lachnospiraceae* ↑, Alpha diversity: =
Ni et al., 2025 [152]	Observational	M/F, 55–75 y, overweight + MetS	Spain	6874	Nut servings per wk (<1×/wk, 1–3×/wk, 3–7×/wk, >7×/wk)	—	Nut intake 3–7×/wk vs. <1×/wk or >7×/wk: lower decline in global cognitive function. Nut intake 1–3×/wk vs. <1×/wk: lower decline in attention.	Overall nut consumption: (+) beta diversity. 3–7×/wk nut consumption: alpha diversity ↑, (+) *Lachnospiraceae UCG-004/Oscillobacter/Roseburia*. >7×/wk nut consumption: (-) *Phascolarctobacterium/Parvimonas/Ruminococcus*. *Lachnospiraceae UCG-004*: (+) GCF/attention.
Soveid et al., 2024 [153]	Cross-sectional	Healthy F, 18–50 y, BMI 20–35, luteal phase	Iran	91	—	—	Plant-protein consumption: (-) depression/anxiety/psychological distress. Animal-protein consumption: (+) psychological distress High plant-protein intake: FFMI ↓. Low plant-protein intake: DBP ↑.	Plant-protein intake: (-) Firmicutes/Firmicutes-Bacteroidetes ratio. Animal-protein intake: (-) *Prevotella*.

Positive associations indicated by (+), negative associations by (-), upregulation/increase by ↑, downregulation/decrease by ↓, no changes by =. When there are differential effects between groups, group names are included in the results.

Abbreviations: DBP, diastolic blood pressure; FFMI, fat-free mass index; GCF, global cognitive function; MetS, metabolic syndrome; PR, picture recognition; RCT, randomized controlled trial; RT, reaction time; RVIP, rapid visual information processing.

## Results

### Body composition

Of the 23 papers that investigated the effects of plant-based dietary interventions on any measurement of body composition, while also reporting on microbial composition or functionality (Table 1), 2 papers described the same study and are therefore combined [121,122]. Many of the included studies compared whole-grain diets to refined-grain intake within omnivorous diets, of which only 1 study showed a significant decrease in body weight in adults at risk of metabolic syndrome (MetS) after 8 wk of ad libitum whole-grain consumption [132]. Three other randomized controlled trials (RCTs) comparing whole-grain to refined-grain consumption, with sample sizes between 33 and 68 adults and lasting 6 to 8 wk, were not able to show differential effects on body composition in both isocaloric [126,137], and ad libitum [118], designs, although total fiber intake was negatively associated with body fat percentage in one of these studies [118]. These studies showed a positive link between whole-grain consumption and Prevotella, with an opposite correlation with refined-grain intake, as depicted in Table 1 [132,137]. Similarly, SCFA production and improvements in body composition were positively associated with *Prevotella* abundance after ad libitum whole-grain consumption in another 6-wk RCT in healthy individuals with overweight [125]. More specifically, *Prevotella* abundance predicted the effects of whole-grain intake, showing greater weight loss in individuals with higher baseline *Prevotella* abundance after whole-grain compared with refined-grain intake [125]. *Faecalibacterium prausnitzii,* another SCFA-producer, was also positively associated with dietary fiber intake, increasing after whole grain consumption, and decreasing after refined-grain intake, whereas *Bacteroides thetaiotaomicron* abundance showed an opposite effect [132]. Two isocaloric studies, aimed at weight maintenance, showed no difference in body composition or energy intake after whole-grain and refined-grain intake [130,135]. However, whole-grain consumption increased energy expenditure and protected against intrahepatic lipid accumulation, whereas fecal SCFA and microbial diversity decreased after refined-grain consumption [130,135].

Three months of germinated brown rice consumption, compared with white rice consumption, reduced body fat percentages in an ad libitum RCT comparing germinated brown rice, germinated black rice, and white rice as part of their regular diet in 68 individuals with T2D and hyperlipidemia [139]. Besides, white rice consumption increased *Clostridium sensu stricto 1* and decreased *Veillonella* abundance compared with germinated brown rice, whereas *Alloprevotella* abundance increased after both germinated black and brown rice compared with white rice consumption [139].

Peanut consumption, evaluated in a 12-wk RCT in 209 adults at risk of MetS, decreased body weight and WC compared with isocaloric rice bar consumption [138]. Similarly, a large cross-sectional study investigating regular nut consumption in ∼3000 Chinese individuals found lower WC, WHR, android fat mass and android-gynoid fat mass ratio, and higher gynoid fat mass in individuals with higher nut consumption compared with low-consuming individuals [136]. However, another RCT testing a nut mixture compared with energy-matched salted potato chips in healthy young adults showed no changes in body composition after 3 wk [134]. Even though the cross-sectional study showed a positive relation between nut consumption and alpha diversity [136], neither RCT showed changes in the gut microbiome [134,138]. In addition, alpha diversity was negatively associated with WC, WHR, relative android fat mass, and android/gynoid ratio, and positively associated with relative gynoid fat mass [136].

Both, 8 wk of almond consumption compared with regular biscuits in individuals with (elevated risk of) T2D [121], and 3 wk of Ajwa date compared with maltodextrin consumption (sugar-content matched) in healthy adults [127], did not alter body composition. However, almond consumption led to increased microbial richness, evenness, and diversity compared with biscuit ingestion, primarily driven by increased abundances of *Ruminococcaceae* spp. [122], whereas date consumption did not affect the microbiome.

We were further able to include studies comparing plant and animal (protein) sources within a whole food matrix. A 4-wk RCT in 113 Australian elderly adhering to 1 of 4 different diets (omnivorous high-fat, omnivorous high-carbohydrate, semivegetarian high-fat, and semivegetarian high-carbohydrate) showed decreased body weight and fat mass after the well-controlled ad libitum dietary intervention. The vegetarian diets induced the greatest weight loss and increase in plasma acetate, whereas the omnivorous, high-carbohydrate diet increased alpha diversity, which in turn was negatively associated with weight loss [131].

When comparing vegetable, animal, and whey protein sources, all within a very low-caloric ketogenic diet (VLCKD), body weight was significantly reduced after vegetable and whey protein ingestion, but not after animal-protein consumption in 48 individuals with metabolic disturbances and overweight [120]. Additionally, both WC and HC decreased in the vegetable-protein group, whereas whey protein only decreased WC, and animal-protein sources solely contributed to lower HC [120]. Firmicutes abundance decreased in all groups, potentially a result of the VLCKD [120]. Animal and whey protein significantly increased relative Bacteroidetes and Proteobacteria abundances, whereas vegetable protein did not [120].

In line with these anthropometric results is a study comparing 200 g of daily red meat consumption to a daily intake of 40 g of fiber and minimal red meat consumption in an isocaloric manner showed that only the latter diet was able to reduce body weight and fat significantly after a 3-wk crossover trial in healthy adults, accompanied by increased microbial diversity [128]. However, isocalorically replacing animal-protein intake with black soymilk consumption in 16 Taiwanese individuals with prediabetes did not induce any body composition changes [124], although *Butyricicoccus* and *Rothia* abundances were significantly increased after 4 wk of replacement [124].

Burns et al. [123] compared plant-based, fasting-mimicking diets with either low- and high-protein content to a weight-maintaining, habitual omnivorous diet in healthy, normal weight adults. Both intervention diets reduced lean and fat mass, whereas the control group remained weight stable. Furthermore, the high-protein diet, compared with control, increased visceral adipose tissue content, alpha diversity and Firmicutes abundance, whereas the low-protein diet reduced Firmicutes abundance compared with control, without affecting alpha diversity [123].

Another RCT tested a fiber mixture of potato fiber and sugar beet pectin compared with isocaloric placebo, whereas both groups adhered to a high-protein, partially plant-based diet in individuals with overweight/obesity at increased risk of developing T2D [129]. In accordance with the study design, both groups remained weight stable after 12 wk, and no changes in body composition or visceral fat depots were observed, nor in microbial composition or functionality, except for a slight increase in plasma isovalerate in the control group [129]. The last prospective single-arm trial evaluated a 6-d lifestyle intervention, where only entirely whole food, plant-based meals were provided, without caloric restrictions, but minimal salt, sugar, and oil intake. The authors report increased body weight and decreased WC and HC, besides increased alpha diversity and the abundance of butyrate-producing bacteria [117].

When linking dietary patterns to microbial composition in young adults, a negative association was found between Bacteroidetes abundance and fiber, protein, carbohydrate, and total energy intake in people with a healthy BMI, whereas these correlations were absent in people with elevated BMIs [133]. Finally, lower fat-free mass was found in Iranian women consuming an unhealthy plant-based diet compared with healthy plant-based diet consumption based on the *Plant-based dietary index* [119], which rates plant-based products on their healthiness [154]. In this observational study, *Prevotella* abundance was positively linked to plant-based diets in general, whereas Firmicutes and *Akkermansia muciniphila* abundance were linked to healthy plant-based diets [119].

Overall, plant-based, whole-grain products seem to have either a positive or no effect on body composition, whereas no negative results of plant-based products and diets on anthropometrics were found in the included studies. However, some studies concluded that animal-derived products negatively impacted body composition [120,128,131]. Notably, some studies report on rather short intervention periods (e.g., 6 d and 3 wk), which are generally too short to draw robust conclusions on changes in anthropometrics and microbial composition. Overall, SCFA-producing bacteria and diversity indices are beneficially affected by plant-focused, whole-grain diets, while being associated with improved body composition. However, due to the lack of data on microbial metabolites such as SCFA, no conclusions on the underlying microbial mediators inducing systemic alterations can be drawn [118,122,125,127,128,132,139].

### Glucose homeostasis

Glucose homeostasis and the (patho)physiology of T2D can, apart from changes in body composition, be affected by many dietary processes, as has been described extensively [26,28,29,[155], [156], [157], [158]]. Although the effects of dietary fiber-derived SCFA are established, benefiting systemic insulin sensitivity and inflammation [26,28,29,158], the effects of proteins on glucose homeostasis are more ambivalent [26,115]. Due to lower digestibility and absorption of plant-proteins, animal proteins generally induce higher incretin secretion, including GLP-1 and GIP, leading to increased insulin secretion and insulin sensitivity [115]. Plant-based proteins, however, have been shown to decrease postprandial insulin responses compared with animal-derived proteins, and reduce HbA_1c_, fasting glucose, and fasting insulin concentrations [99,[159], [160], [161], [162]]. Because gut dysbiosis and increased gut permeability, caused by TMAO, LPS, and p-cresol, among others, have been linked to reduced insulin sensitivity, potentially induced by proteolytic fermentation [163], there is a need for future clarification of these processes.

We included 16 articles investigating the effects of plant-based diets on glucose homeostasis that also reported microbial data (Table 2). Most of the studies were conducted in metabolically compromised populations and investigated the effects of a plant-based product that either replaced a part of a regular diet or was implemented as a dietary additive. Galié et al. [143,144] compared 50 g of nut consumption compared with the Mediterranean diet with low-nut consumption in 44 adults with MetS in a 2-mo, isocaloric RCT. The MD, but not nut consumption, led to significant decreases in glucose, insulin, and HOMA-IR values, and increases in plasma SCFA concentrations [143,144]. Neither alpha and beta diversity, nor the Firmicutes/Bacteriodetes ratio was altered, whereas *Lachnospiraceae NK4A316* and *Ruminococcaceae* spp. were enriched after 8 wk of MD [144]. In this study, HOMA-IR was negatively associated with *Lachnospiraceae NK4A136* abundance [143]. Despite the lack of nut-related effects in this study, in another RCT, walnut consumption, compared with an isocaloric, nut-free placebo in 10 individuals with obesity was found to improve glucose and insulin responses, and lower fecal BCFAs, without affecting bacterial composition [149].

Medina-Vera et al. [145] assessed the effect of combining several plant-proteins compared with an isocaloric casein-maltodextrin mixture in 53 adults with overweight and pharmacologically-treated T2D, showing improved HbA_1c_ concentrations after 3 mo in the intervention group. This was accompanied by increased abundances of *Faecalibacterium prausnitzii*, *Bifidobacterium longum*, and *Akkermansia muciniphila*, and reduced *Prevotella copri* abundance [145].

Basciani et al. [120] reported a 45-d weight-loss trial after VLCKDs with 3 different protein sources (animal, whey, and vegetable), showing decreased insulin and HOMA values after all protein sources, likely induced by the VLCKD. Similarly, Firmicutes abundance decreased in all groups, whereas Bacteroides only increased in the animal and whey group [120]. Wu et al. [150] compared animal compared with plant-protein intake within a calorie-restricted diet in a 16-wk RCT in 127 Chinese individuals with prediabetes and also showed positive effects of the calorie restriction in the overall population, but without any differential effects on glucose homeostasis between the groups, except for a greater, temporal decrease in HbA_1c_ in the plant-protein group. Similar to Basciani et al.’s results, both calorie-restricted diets induced changes in microbial composition, although more pronounced in the plant-protein group, evidenced by increased *Bifidobacterium* and *Roseburia* and decreased *Bacteroides* abundance after legume consumption, among others [150]. Besides, legume consumption increased plasma SCFA concentrations, without affecting fecal SCFA concentrations, although the latter were positively associated with HbA_1c_ in the intervention group [150]. Plasma acetate, in turn, was negatively associated with cardiometabolic markers, including HbA_1c_ [150]. A comparison between black soy milk and pork consumption did not indicate differential effects on cardiometabolic markers either, although soy milk increased *Butyricicoccus* and *Rothia* abundance, as described before [124].

Several RCTs described the effects of fiber-rich products [139,129,141,148]. Su et al. [148] found improved HbA_1c_ and fasting glucose concentrations in 16 patients with T2D after a combination of whole grains with prebiotics and probiotics, accompanied by a reduction in alpha diversity. Besides, after 90 d of this dietary intervention, the most abundant species had shifted from *Bacteroides* to *Prevotella,* accompanied by increases in relative *Bifidobacteria, Blautia, Acidaminococcus,* and *Pseudomonas* abundances*,* and decreases in *Bilophila, Oscillospira, Roseburia,* and *Ruminococcus* abundances [148]. These data must be interpreted with caution, due to the combination of whole grains with prebiotics and probiotics provided in the intervention [148]. However, regardless of the intervention, *Bifidobacterium* and *Lactobacillus* abundances showed negative associations with several cardiometabolic markers, including fasting blood glucose concentrations, HbA_1c_, blood pressure, LDL, and BMI [148]. Surprisingly, one 12-wk RCT, comparing a fiber mixture compared with isocaloric maltodextrin in individuals with overweight/obesity at elevated risk of T2D, with both groups receiving a protein-rich, partially plant-based diet, showed improvements in insulin sensitivity in the placebo group. Although the placebo group, consuming only the plant-protein-rich diet, improved, insulin sensitivity in the fiber seemed to decrease, without affecting fasting glucose, insulin or HbA_1c_ [129]. Both groups did not show changes in microbial composition or functionality, besides an increase in plasma isovalerate in the placebo group [129].

Germinated brown rice, compared with refined white rice in a 12-wk RCT in 112 individuals with T2D lowered fasting glucose and HbA_1c_ concentrations [141], which was in line with results by Zhao et al. [139] showing decreased fasting blood glucose concentrations after both germinated black and brown rice compared with white rice after 3 mo in 68 individuals with T2D. However, these studies reported different microbial outcomes [139,141]. Ding et al. [141] found that germinated brown rice increased relative abundances of *Bifidobacterium, Actinomycetes,* and *Pasteurellales* besides increasing fecal SCFA production, whereas Zhao et al. [139] mainly showed increased *Alloprevotella* abundance after germinated rice adherence, with a white rice-induced increase in *Clostridium sensu stricto 1*. Moreover, germinated brown rice, compared with germinated black rice, increased *Fusobacteria* abundance, without any effects on microbial metabolites [139].

Another study, comparing fava bean to buckwheat, showed that only fava bean consumption significantly reduced fasting blood glucose concentrations in 20 healthy United Kingdom residents after 7 d [146]. Although buckwheat consumption, although not affecting glucose metabolism, decreased *Roseburia*, *Anaerostipes*, *Bifidobacterium*, and *Dorea* species, fava bean consumption decreased *Lactobacillus* abundance and increased *Coprococcus* and *Bifidobacterium* abundance [146]. Neither intervention affected microbial metabolites [146].

Two observational studies show a positive association between healthy, plant-based dietary patterns and improved glucose metabolism [140,147]. Besides, the larger cohort study with a follow-up of 4–8 y showed that protein intake was significantly associated with prediabetes and T2D prevalence, almost fully explained by animal-protein intake [140]. In this study, fat intake was positively associated with alpha diversity, whereas carbohydrate intake showed a negative association, and protein showed no association [140]. Both total fiber and plant-protein intake were significantly associated with beta diversity, whereas total protein and animal-protein intake showed no associations [140]. Stefani et al. [147] only investigated *Bifidobacterium* abundance, but found no associations with dietary intake.

Although the above findings indicate primarily positive effects of plant-derived product ingestion, 2 RCTs, both performed in ∼50 adults with MetS >8 wk, did not show any changes in indices of glucose metabolism after comparing different types and amounts of whole grains [132,142]. However, they reported increased *Faecalibacterium prausnitzii* and *Prevotella copri* abundances, while decreasing *Bacteroides thetaiotaomicron* abundance after increased whole-grain consumption, without affecting SCFA concentrations [132]. No metabolic differences were found between whole-grain rye and whole-grain wheat, although microbial composition seemed to be negatively affected by rye intake, showing lower abundances of *Bifidobacteria*, *Lachnospira*, and *Butyricicoccae* [142].

In summary, of the 15 individual studies included, 12 found positive results, mostly in fasting glucose or HbA_1c_ concentrations, after increased intake of plant-based whole foods or additives. More importantly, none of the studies reported adverse effects on glucose homeostasis after adding or increasing plant-derived product intake, except 1 study, showing benefits of a high-protein, partially plant-based diet, but a negative effect of combining a specific fiber mixture with increased plant-protein consumption. Microbial composition can be optimized through these plant-focused dietary interventions, increasing microbial diversity and upregulating abundances of microbes such as *Bifidobacterium, Faecalibacterium prausnitzii, Akkermansia muciniphila, Lachnospiraceae,* and *Prevotella*.

### Neurocognition

Although the link between the gut and metabolic health has been studied for decades, scientific interest in the gut-brain axis has increased more recently. Unfortunately, not much is known about the microbiome-mediated effects of plant-based products on neurocognition. Neurocognition and brain health are rather broad concepts, including general mental well-being, but also cognitive diseases such as Alzheimer’s Disease. So far, it has been shown that some specific nuts positively impact neurocognitive performance and body weight [164,165]. The role of plant-based diets and the microbiome herein, however, is unknown, although dietary fibers and SCFA have been linked to improved gut-brain axis, satiety, hormonal responses, memory, and gut barrier function, and lowered (neuro)inflammation, which may affect the development of specific diseases [[166], [167], [168]]. However, data remain contradictory, as the prevalence of depressive symptoms was higher in vegetarians compared with nonvegetarians in 1 study, whereas another study showed beneficial associations between mood disorders and vegan diet adherence [166,169,170]. This highlights the complex interplay between diet and neurocognitive effects, while also exposing the fact that not all plant-based diets are comparable in terms of their impact on human well-being.

Three studies were included reporting neurocognitive and microbial effects after plant-based product ingestion (Table 3). The only included RCT investigated the effects of mixed-nut consumption in healthy adults with a BMI of 18.5–30 kg/m^2^ [151]. Significant improvements were found in memory, executive function, and attention after 28 d of nut consumption, comparing a combined 30 g of walnuts, almonds, and hazelnuts to a placebo [151]. Additionally, *Lachnospiraceae* abundance increased in the intervention group, but not in the placebo group, without changes in alpha diversity in both groups [151].

Furthermore, we encountered 2 cross-sectional studies. One study, investigating the relationship between plant-protein consumption and neuropsychological functioning in 91 Iranian women found that higher plant-protein consumption was associated with lower fat-free mass, as well as lower occurrence of depression, anxiety, and psychological distress [153]. Contrarily, animal-protein intake was associated with a higher risk of psychological stress [153]. Similar positive effects of nut consumption were found in a large longitudinal cohort with >6 y of follow-up in 6874 Spanish individuals, showing that moderate nut consumption was associated with lower cognitive decline and a slower decline in attention [152]. Besides, moderate nut consumption correlated with higher microbial diversity and *Roseburia* abundance, among others [152]. Plant-based protein consumption was negatively associated with Firmicutes abundance and the Firmicutes/Bacteroidetes ratio, whereas animal-protein intake was negatively associated with *Prevotella* abundance [153]. Moreover, *Lachnospiraceae UCG-004* was positively associated with greater cognitive function [152].

Similar to the positive effects on body composition and glucose homeostasis, plant-product ingestion seems to aid neurocognitive performance and preservation, contributing to a sustainable neuropsychological status. Even though a small number of studies have been performed, evidence suggests that some bacterial taxa can be altered through plant-based dietary habits, which in turn might mediate neuroprotective benefits.

## Discussion and Perspectives

To our knowledge, this review is one of the first to illustrate the crosstalk between plant-focused diets, the microbiome, and their potential to improve metabolic health in multiple domains. Because the microbiome has only been considered as a key mediator in many regulatory processes during the last decades, publications on microbiome-mediated effects of plant-based diets on host health are scarce. Most studies have been published in the last decade, providing up-to-date knowledge using state-of-the-art methods. Overall, we were able to identify some species and taxa that seemingly contribute to metabolic improvements, of which abundances were altered through dietary interventions. Not all studies reported their results of the microbiome in a comparable manner, but many reports include changes in either microbial diversity, richness, bacterial abundance, or substrate production, allowing us to draw some general conclusions from these studies.

First, independent of the outcome parameter, plant-based products or diets either have a beneficial or no differential effect on metabolic processes, glucose homeostasis, neurocognition, or body composition. These results are in line with other recent studies, showing the potential of vegan and vegetarian diets to tackle common metabolic diseases, such as T2D, CVD, and obesity [16,18,36,116,[171], [172], [173]]. Dietary fibers and phytochemicals play a pivotal role in these advantages, although the extent to which fibers reach the colon and are fermented, however, heavily depends on the type of fiber and the food matrix [58]. And even though plant-proteins are generally less digestible, potentially resulting in higher colonic contents, and proteolytic fermentation has been linked to metabolic deterioration, we did not see any evidence of negative effects on host health. Potential explanations might lie in the fact that plant-proteins are likely to be coingested with other beneficial dietary components (i.e., dietary fiber and polyphenols), fermentation of plant-proteins, compared with animal proteins, yields more beneficial microbial compounds, plant-proteins are fermented to a lesser extent than animal proteins due to their chemical structure, or that differences in the amino acid content yield different substrates [25,26,174,175]. Moreover, the fact that plant-based products generally contain less protein compared with animal-derived sources might contribute to these findings, even though these differences can be overcome by combining different plant-protein sources [176,177]. However, it must be noted that plant-based diets are diverse, and the great variety in the composition of these diets varies in terms of healthiness. Publication and researcher bias may be due to the fact that experiments or interventions are conducted catering to expectations, leaving a gap for thorough RCTs on the effects of unhealthy plant-based diets. Hence, concluding that all plant-based diets contribute to metabolic benefits is impossible, especially given the data regarding refined-grain and white rice consumption compared with their healthier alternatives. Future studies on the microbiome-host-metabolic effects after more unhealthy plant-based diets are needed.

Second, most of the data regarding microbial composition or functionality move in a similar direction or show a consistent pattern of microbial changes. It has been shown that VLCKDs reduced Firmicutes abundances, especially when combined with vegetable-protein intake, which was accompanied by metabolic benefits [120]. Higher fiber intake was, in turn, associated with lower Bacteroidetes and not Firmicutes abundance [133]. However, with more in-depth technologies, deeper identification beyond the phylum level is possible and warranted to accurately address beneficial or detrimental effects of dietary interventions, combined with analyzing microbial functionality. We identified many species associated with increased plant-based diet consumption with diverse effects on host metabolism. *Prevotella*, a prominent Bacteroidetes SCFA-producer, has been found increasingly prevalent after whole-grain, plant-derived product ingestion in many of the studies [132,137,125,139,119], and is known to contribute to beneficial effects on body composition and glucose homeostasis. However, the association between *Prevotella* abundance and neurocognitive aspects is more difficult to pinpoint [178]. Although our results show only a negative association with animal-protein consumption, other literature found *Prevotella* to be less abundant in patients with Parkinson’s disease compared with healthy controls [178,179], whereas on the other hand, its abundance is associated with cognitive impairment and decline [178,179]. Potential beneficial Firmicutes species, including *Lachnospiraceae*, *Clostridiaceae*, *Ruminococcaceae*, *Roseburia,* and *Faecalibacterium prausnitzii*, for example, were found in higher abundances after plant-based or whole-grain interventions, and were often accompanied by improved metabolic health, body composition, and neurocognitive traits [132,117,143,144,145,148,151,152]. Many of these beneficial genera and species may be involved in SCFA production [29,[180], [181], [182]]. Besides the 2 prominent phyla, Actinobacteria, and especially the *Bifidobacteria,* have also been linked to metabolic improvements in many of the reviewed studies [29], which may be upregulated through a plant-based dietary pattern [54,141]. However, as is the case with metabolic outcomes, the variation among plant-based diets may result in differential microbial outcomes, as it has been found that *Faecalibacterium* and *Roseburia*, for example, are more abundant in plant-based or (lacto-ovo) vegetarian diets, but less abundant in vegan diets [43]. Whether metabolic benefits occur simultaneously through different processes or as a consequence of increased abundance remains speculative [178,179,[182], [183], [184], [185], [186], [187]]. Although the reviewed studies showed changes in the abundance of SCFA-producers, most studies did not report alterations in SCFA. Generally, however, even though SCFA production might not necessarily be altered, no adverse effects were described after upregulation of these species. Furthermore, even though many of the aforementioned species are associated with metabolic improvements, their association with neurocognition often remains more ambivalent. Although some species, including *Faecalibacterium prausnitzii* [183,184], Ruminococcaceae [180], and *Bifidobacterium* [185,186], generally show beneficial associations with neurocognition, some species, such as *Roseburia* and *Lachnospiraceae,* have been linked to both positive [181,187], and negative [181,182], associations with neurocognition and neurobiological diseases. Although, based on the above, many species can be classified as mainly beneficial, further research and deeper phenotyping of these microbes is essential to understand their interaction with both metabolic and neurocognitive processes.

Third, the gut microbiome differs per individual, but more importantly, between metabolic phenotypes, potentially affecting the impact of dietary interventions [29,37,188]. Christensen et al. [125], for example, showed that people with lower *Prevotella* abundance at baseline were at a disadvantage to lose body weight with whole-grain consumption, and, surprisingly, were only able to decrease their waist circumference after refined-grain ingestion. Contrarily, higher baseline *Prevotella* abundance contributed to weight loss after a whole-grain diet [125]. Other studies have reported similar associations with baseline *Prevotella* abundance, showing that individuals with a higher *Prevotella*/*Bacteroides* ratio either lose more weight or are protected from weight gain on a high-fiber diet [188,189]. Furthermore, it has been found that the microbiome of people with (pre-)T2D is different in both composition and its capacity to ferment dietary substrates compared with healthy controls [30,145]. Besides these associations between enterotype and metabolic effects, individuals with high *Prevotella* abundance have also been found to show different emotion regulatory activity and lower hippocampal activity compared with the *Bacteroides* enterotype [190]. This underlines the need to investigate further how individual baseline (metabolic) phenotypes can be incorporated into therapeutic and preventive strategies. Defining the gut microbiome before starting any (dietary) intervention or prebiotic/probiotic treatment might thereby contribute to optimized results by re-establishing gut microbial symbiosis through specifically targeting underrepresented microbial species, while also optimizing microbial metabolite production, for example [191]. Establishing causality, however, remains difficult studying diet–microbe–host metabolism interactions. Even though it has been shown that there are fundamental (microbial) differences between metabolic phenotypes, it is not yet clear whether the microbiome reacts differently between individuals due to their phenotype, vice versa, or both [29,37,188]. Moreover, intrinsic differences in metabolic processes might affect the individual microbiome and, consequently, metabolic profile [29,37,188]. Besides, even though baseline microbial composition might serve as a start to determine intervention efficacy, at this point, the human gut remains rather complex, with differences in microbial composition in each segment, troubling the incorporation of baseline microbial composition into nutritional strategies. Nevertheless, studies making use of fecal microbial transplantation or other microbiome-modulating interventions that alter gut microbial composition have been able to induce metabolic consequences [1,74,148,[192], [193], [194], [195], [196], [197], [198], [199]]. Besides, the fact that microbial disturbances may be present in early stages of disease development contributes to the likely causal role of microbiome-related alterations affecting host metabolism [1,82,[199], [200], [201]].

There are some limitations to this review and the studies included, providing future research opportunities. Although we show associations between plant-based dietary components, microbial composition, and effects on host health, drawing definitive conclusions on causal relationships between microbial communities, their functionality, and changes in host metabolism is impossible due to the great variety in study designs. Besides, including studies from many different countries around the world leads to heterogeneity in background dietary intake and (epi)genetics, but also in sample collection, storage, and analyses. Furthermore, the gut microbiome can be analyzed and described in numerous ways, with some studies only reporting data on the phylum level, whereas others analyzed microbial subspecies or metabolites, with data obtained through either 16S RNA sequencing or more detailed whole-genome qPCR.

Apart from the commonly studied products such as whole grains, nuts, and legumes, more modern, sustainable meat alternatives are finding their way into our diet. Algae and fungal proteins, for example, are increasingly recognized for their benefits and low environmental footprint. Unfortunately, none of the included studies investigated the effects of these products, warranting future research on these sustainable alternatives in light of human health. Similarly, as this review focused on plant-based interventions, including whole-grain products but also healthy plant-based products such as germinated rice, we did not include studies specifically comparing different plant-focused diets such as pesco-ovo-lacto–vegetarian, the Mediterranean or Atlantic diets, each also known for their metabolic benefits and sustainability compared with the Western diet. Finally, even though we encountered a variety of studies investigating the effects of plant-based diets on body composition and glucose homeostasis, the fact that only 3 studies reporting on neurocognition were included, and only one of which was an RCT, highlights the underexplored area of gut-brain communication in relation to plant-based interventions.

Overall, we can conclude that adhering to plant-based, whole-grain diets aids in maintaining or improving (metabolic) health. Besides, upregulation of certain bacterial species, such as *Bifidobacteria*, *Prevotella*, or *Faecalibacterium prausnitzii*, which are tightly associated with improvements in host health, may appear through plant-based approaches. However, these benefits for the gut microbiome and host are primarily related to the so-called healthy plant-based diets, with limited sugary beverages, sweets, and refined-grain intake. Although unhealthy plant-based diets contain less beneficial products and potentially higher amounts of sugars and refined grains, it may still be hypothesized that unhealthy plant-based diets, containing many plant-derived substrates, such as fibers and phytochemicals, can still induce metabolic advantages over omnivorous diets, which is in need of further exploration.

The reviewed data can be interpreted as supportive of the societal shift to more plant-based product consumption. With respect to dietary protein content, we advocate the use of whole foods and a balanced diet with various (protein) sources to overcome lower digestibility and absorption of plant-based proteins, ensuring sufficient amino acid intake, while preventing deficiencies and related complications such as sarcopenia, one of the most proposed downsides of vegan diets [176,177]. Furthermore, studies on the long-term effects of the consumption of different plant-based dietary patterns on metabolic health, as well as individual variations in response to dietary interventions, are warranted.

## Author contributions

The authors’ responsibilities were as follows – CAJvK: conducted the search, read and summarized all included papers, and wrote the manuscript; CAJvK, TCA: individually assessed the title and abstract to determine eligibility; TCA, EEB: provided supervision, and reviewed and edited the manuscript; EEB: had primary responsibility for the final content; and all authors: designed, read, and approved the final manuscript.

## Funding

This research has been made possible by the support of the Nederlandse Organisatie voor Wetenschappelijk Onderzoek (NWO; The Dutch Research Council) through the ENW-private public partnership fund, the Carbohydrate Competence Center (part of Next Food Collective). The funding partners were not involved in the design of the study, nor in the collection, analysis, and interpretation of the study.

## Conflict of interest

EEB reports having received funding grants from the Dutch Research Council. EEB is project leader in several public–private partnerships between academia and food industry (Sensus, Whole Fiber B.V., Avebe, Nuscience and Agrifirm). All authors confirm they have no conflict of interest.
