# Supplementary material for: Gut Microbiome-Associated Effects of Plant-Based Diets on Glucose Homeostasis, Body Composition, and Cognitive Function: A Scoping Review
Source: Adv Nutr. 2026 Feb 22;17(4):100610. doi: 10.1016/j.advnut.2026.100610 (PMC13000513; doi:10.1016/j.advnut.2026.100610)
Supplement: Multimedia component 1 [file mmc1.docx]

**Gut microbiome-associated effects of plant-based diets on glucose homeostasis, body composition, and cognitive function: a scoping review**

Colin A.J. van Kalkeren et al.

Supplementary materials

# Supplementary material

## Body composition search string

((("Plant-based"[All Fields] AND ("protein s"[All Fields] OR "proteinous"[All Fields] OR "proteins"[MeSH Terms] OR "proteins"[All Fields] OR "protein"[All Fields])) OR ("plant-sourced"[All Fields] AND ("protein s"[All Fields] OR "proteinous"[All Fields] OR "proteins"[MeSH Terms] OR "proteins"[All Fields] OR "protein"[All Fields])) OR ("plant proteins, dietary"[MeSH Terms] OR ("plant"[All Fields] AND "proteins"[All Fields] AND "dietary"[All Fields]) OR "dietary plant proteins"[All Fields] OR ("vegetable"[All Fields] AND "protein"[All Fields]) OR "vegetable protein"[All Fields]) OR "soy"[All Fields] OR ("glutens"[MeSH Terms] OR "glutens"[All Fields] OR "gluten"[All Fields]) OR ("triticum"[MeSH Terms] OR "triticum"[All Fields] OR "wheat"[All Fields] OR "wheat s"[All Fields] OR "wheats"[All Fields]) OR ("cereale"[All Fields] OR "edible grain"[MeSH Terms] OR ("edible"[All Fields] AND "grain"[All Fields]) OR "edible grain"[All Fields] OR "cereal"[All Fields] OR "cereals"[All Fields]) OR ("oryza"[MeSH Terms] OR "oryza"[All Fields] OR "rice"[All Fields]) OR ("barley s"[All Fields] OR "hordeum"[MeSH Terms] OR "hordeum"[All Fields] OR "barley"[All Fields] OR "barleys"[All Fields]) OR ("fabaceae"[MeSH Terms] OR "fabaceae"[All Fields] OR "legume"[All Fields] OR "legumes"[All Fields]) OR ("nuts"[MeSH Terms] OR "nuts"[All Fields]) OR ("Plant-based diet"[All Fields] OR "Plant-based diets"[All Fields])) AND ("body composition"[All Fields] OR "anthropometric"[All Fields] OR "anthropometrical"[All Fields] OR "anthropometrically"[All Fields] OR "anthropometrics"[All Fields] OR "whr"[All Fields] OR "Waist-to-hip"[All Fields] OR "waist to hip ratio"[All Fields] OR "Waist-to-hip"[All Fields] OR "body weight"[All Fields] OR "fat distribution"[All Fields] OR "fat deposition"[All Fields]) AND ("microbiome s"[All Fields] OR "microbiomic"[All Fields] OR "microbiomics"[All Fields] OR "microbiota"[MeSH Terms] OR "microbiota"[All Fields] OR "microbiome"[All Fields] OR "microbiomes"[All Fields] OR "gut microbiome"[All Fields] OR "gut bacteria"[All Fields] OR "intestinal microbiome"[All Fields] OR "gut microbiota"[All Fields] OR "Intestinal microbiota"[All Fields] OR "microbiome-mediated"[All Fields] OR "microbiome-mediated"[All Fields] OR "microbiota-mediated"[All Fields] OR "microbiome-mediated"[All Fields] OR "bacteria"[All Fields])) AND ((humans[Filter]) AND (dutch[Filter] OR english[Filter]) AND (alladult[Filter]))


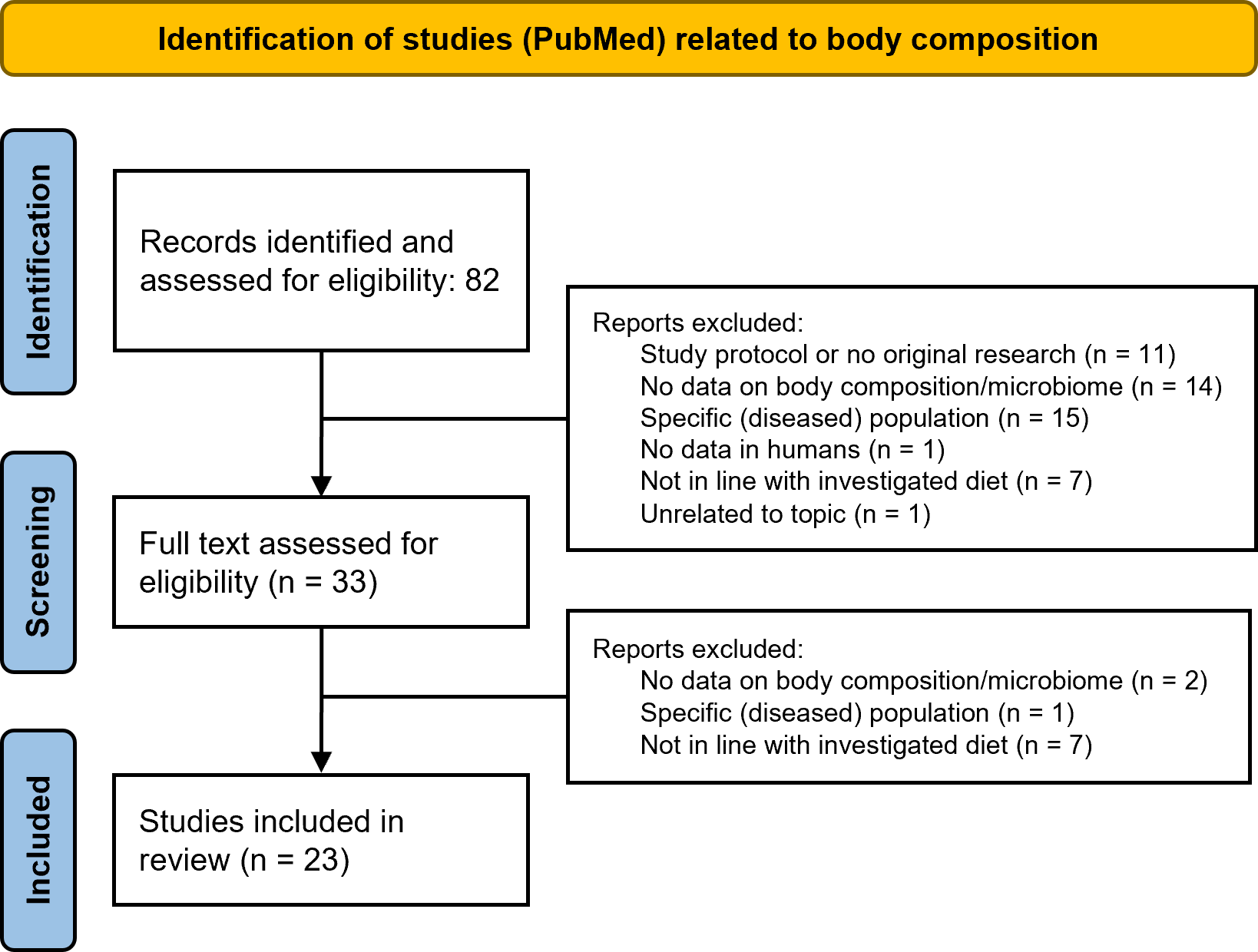


Supplementary Figure 1. PRISMA flowchart of the included studies related to body composition.

## Glucose homeostasis search string

((("Plant-based"[All Fields] AND ("protein s"[All Fields] OR "proteinous"[All Fields] OR "proteins"[MeSH Terms] OR "proteins"[All Fields] OR "protein"[All Fields])) OR ("plant-sourced"[All Fields] AND ("protein s"[All Fields] OR "proteinous"[All Fields] OR "proteins"[MeSH Terms] OR "proteins"[All Fields] OR "protein"[All Fields])) OR ("plant proteins, dietary"[MeSH Terms] OR ("plant"[All Fields] AND "proteins"[All Fields] AND "dietary"[All Fields]) OR "dietary plant proteins"[All Fields] OR ("vegetable"[All Fields] AND "protein"[All Fields]) OR "vegetable protein"[All Fields]) OR "soy"[All Fields] OR ("glutens"[MeSH Terms] OR "glutens"[All Fields] OR "gluten"[All Fields]) OR ("triticum"[MeSH Terms] OR "triticum"[All Fields] OR "wheat"[All Fields] OR "wheat s"[All Fields] OR "wheats"[All Fields]) OR ("cereale"[All Fields] OR "edible grain"[MeSH Terms] OR ("edible"[All Fields] AND "grain"[All Fields]) OR "edible grain"[All Fields] OR "cereal"[All Fields] OR "cereals"[All Fields]) OR ("oryza"[MeSH Terms] OR "oryza"[All Fields] OR "rice"[All Fields]) OR ("barley s"[All Fields] OR "hordeum"[MeSH Terms] OR "hordeum"[All Fields] OR "barley"[All Fields] OR "barleys"[All Fields]) OR ("fabaceae"[MeSH Terms] OR "fabaceae"[All Fields] OR "legume"[All Fields] OR "legumes"[All Fields]) OR ("nuts"[MeSH Terms] OR "nuts"[All Fields]) OR ("Plant-based diet"[All Fields] OR "Plant-based diets"[All Fields])) AND ("diabetes mellitus"[All Fields] OR "type 2 diabetes"[All Fields] OR "T2DM"[All Fields] OR "Type 2 Diabetes Mellitus"[All Fields] OR ("prediabetic state"[MeSH Terms] OR ("prediabetic"[All Fields] AND "state"[All Fields]) OR "prediabetic state"[All Fields] OR "prediabetes"[All Fields] OR "prediabetic"[All Fields] OR "prediabetics"[All Fields]) OR ("Insulin sensitivity"[All Fields] OR "insulin resistance"[All Fields] OR "HOMA-IR"[All Fields] OR "glucose homeostasis"[All Fields] OR "glucose disposal"[All Fields] OR "glucose uptake"[All Fields])) AND ("microbiome s"[All Fields] OR "microbiomic"[All Fields] OR "microbiomics"[All Fields] OR "microbiota"[MeSH Terms] OR "microbiota"[All Fields] OR "microbiome"[All Fields] OR "microbiomes"[All Fields] OR "gut microbiome"[All Fields] OR "gut bacteria"[All Fields] OR "intestinal microbiome"[All Fields] OR "gut microbiota"[All Fields] OR "Intestinal microbiota"[All Fields] OR "microbiome-mediated"[All Fields] OR "microbiome-mediated"[All Fields] OR "microbiota-mediated"[All Fields] OR "microbiome-mediated"[All Fields] OR "bacteria"[All Fields])) AND ((fft[Filter]) AND (humans[Filter]) AND (dutch[Filter] OR english[Filter]) AND (alladult[Filter]))


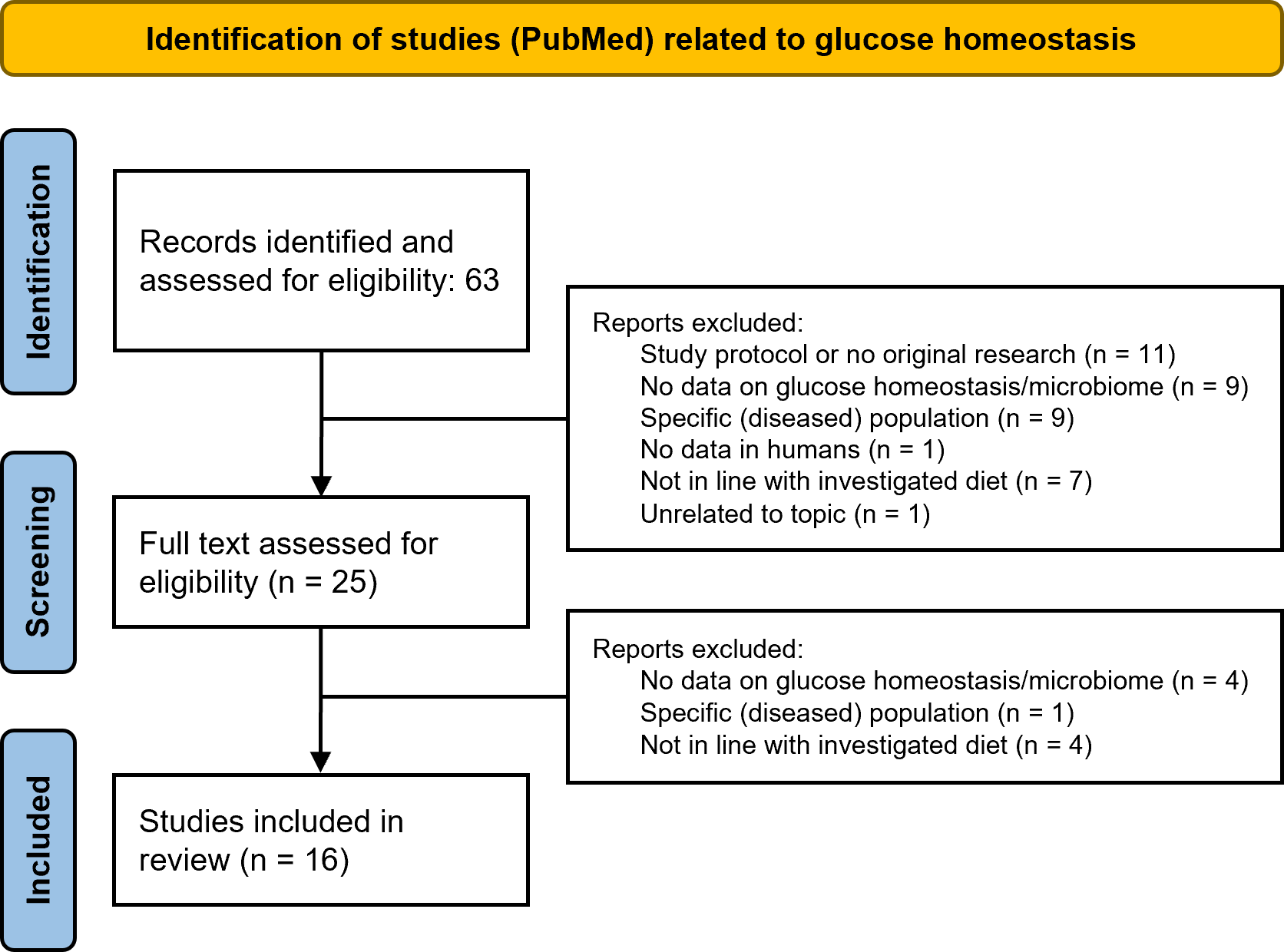


Supplementary Figure 2. PRISMA flowchart of the included studies related to glucose homeostasis.

## Neurocognition search string

((("Plant-based"[All Fields] AND ("protein s"[All Fields] OR "proteinous"[All Fields] OR "proteins"[MeSH Terms] OR "proteins"[All Fields] OR "protein"[All Fields])) OR ("plant-sourced"[All Fields] AND ("protein s"[All Fields] OR "proteinous"[All Fields] OR "proteins"[MeSH Terms] OR "proteins"[All Fields] OR "protein"[All Fields])) OR ("plant proteins, dietary"[MeSH Terms] OR ("plant"[All Fields] AND "proteins"[All Fields] AND "dietary"[All Fields]) OR "dietary plant proteins"[All Fields] OR ("vegetable"[All Fields] AND "protein"[All Fields]) OR "vegetable protein"[All Fields]) OR "soy"[All Fields] OR ("glutens"[MeSH Terms] OR "glutens"[All Fields] OR "gluten"[All Fields]) OR ("triticum"[MeSH Terms] OR "triticum"[All Fields] OR "wheat"[All Fields] OR "wheat s"[All Fields] OR "wheats"[All Fields]) OR ("cereale"[All Fields] OR "edible grain"[MeSH Terms] OR ("edible"[All Fields] AND "grain"[All Fields]) OR "edible grain"[All Fields] OR "cereal"[All Fields] OR "cereals"[All Fields]) OR ("oryza"[MeSH Terms] OR "oryza"[All Fields] OR "rice"[All Fields]) OR ("barley s"[All Fields] OR "hordeum"[MeSH Terms] OR "hordeum"[All Fields] OR "barley"[All Fields] OR "barleys"[All Fields]) OR ("fabaceae"[MeSH Terms] OR "fabaceae"[All Fields] OR "legume"[All Fields] OR "legumes"[All Fields]) OR ("nuts"[MeSH Terms] OR "nuts"[All Fields]) OR ("Plant-based diet"[All Fields] OR "Plant-based diets"[All Fields])) AND ("brain"[MeSH Terms] OR "brain"[All Fields] OR "brains"[All Fields] OR "brain s"[All Fields] OR ("stress"[All Fields] OR "stressed"[All Fields] OR "stresses"[All Fields] OR "stressful"[All Fields] OR "stressfulness"[All Fields] OR "stressing"[All Fields]) OR ("neurocognition"[All Fields] OR "neurocognitive"[All Fields] OR "neurocognitively"[All Fields]) OR ("neurocognition"[All Fields] OR "neurocognitive"[All Fields] OR "neurocognitively"[All Fields])) AND ("microbiome s"[All Fields] OR "microbiomic"[All Fields] OR "microbiomics"[All Fields] OR "microbiota"[MeSH Terms] OR "microbiota"[All Fields] OR "microbiome"[All Fields] OR "microbiomes"[All Fields] OR "gut microbiome"[All Fields] OR "gut bacteria"[All Fields] OR "intestinal microbiome"[All Fields] OR "gut microbiota"[All Fields] OR "Intestinal microbiota"[All Fields] OR "microbiome-mediated"[All Fields] OR "microbiome-mediated"[All Fields] OR "microbiota-mediated"[All Fields] OR "microbiome-mediated"[All Fields] OR "bacteria"[All Fields])) AND ((fft[Filter]) AND (humans[Filter]) AND (dutch[Filter] OR english[Filter]) AND (alladult[Filter]))


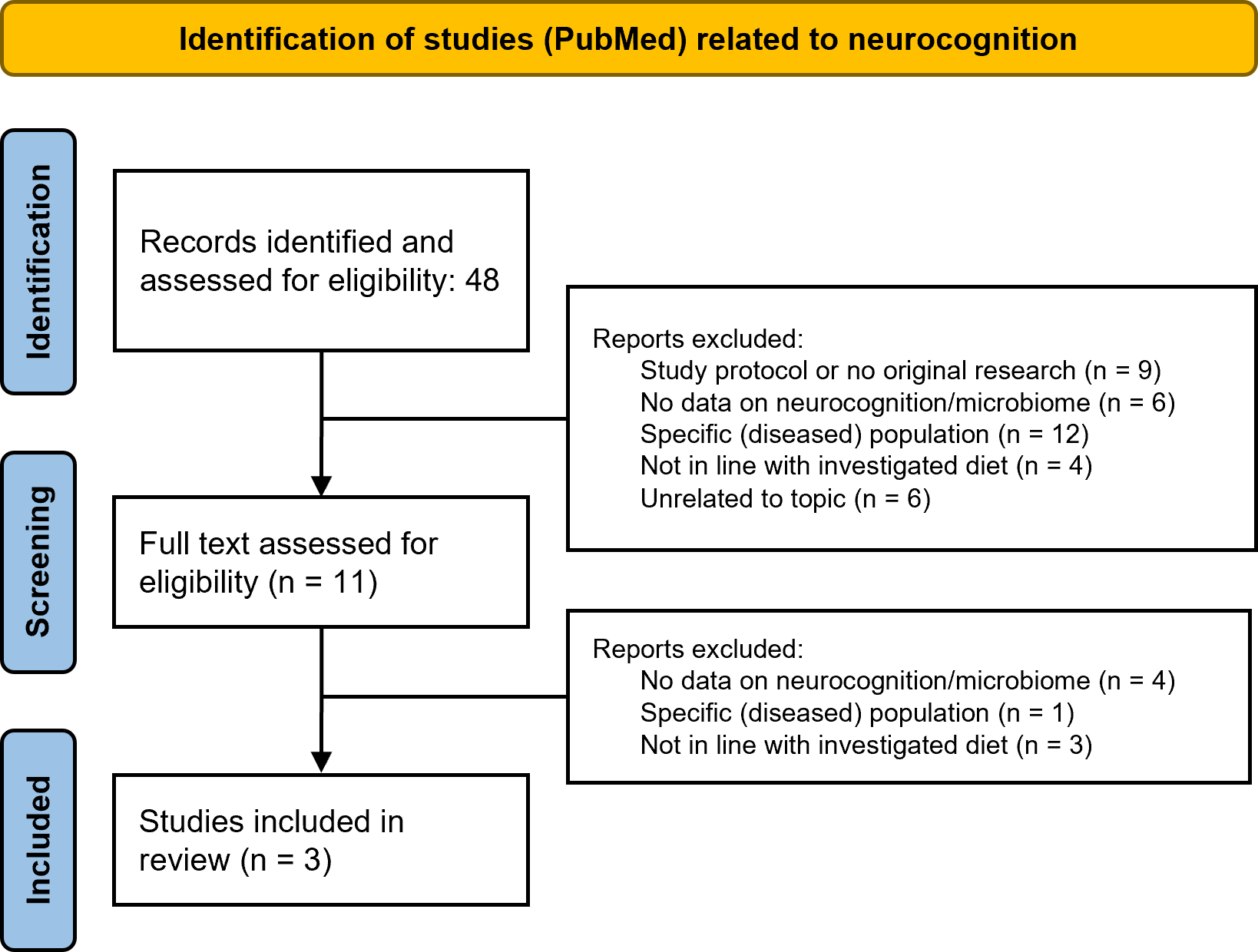


Supplementary Figure 3. PRISMA flowchart of the included studies related to neurocognition.
